# 

**Published:** 1890-02

**Authors:** 


CONTENTS:

PAGE

ORIGINAL ARTICLES.—
- Odontomes. By J. Bland Sutton, F.R.C.S . . . . . . . . . 89
- Hints to Explorers and Naturalists in the Field. By R. W. Shufeldt, M.D., C.M.Z.S. . . . . . . . . 98
- A Case of Lupus. By Langdon Frothingham, M.D.V. . . . . . . . . 103
- Sarcoma. By Dr. R. C. Mason. . . . . . . . . 106
- Age of the Horse, Ox, Dog, and other Domestic Animals. By R. S. Huidekoper, M.D., Veterinarian. . . . . . . . . 107

EDITORIAL DEPARTMENT.—
- Veterinary Legislation . . . . . . . . 113
- Tuberculosis . . . . . . . . 115
- Meeting of United States Veterinary Association . . . . . . . . 117
- Corrigenda . . . . . . . . 117

CASES, SELECTIONS, TRANSLATIONS.—
- Glycerine in Veterinary Practice. By Dr. J. Coates, M.D., D.V.S. . . . . 118
- Physical Condition of Horses for Military Purposes. By George Fleming, C.B., LL.D., F.R.C.S. . . . . . . . . 120
- Remounts, U. S. Army. By Lieutenant W. Baird, 6th Cavalry . . . . . . 132
- A New Cattle Pest . . . . . . . . 137
- Riding Feats . . . . . . . . 137
- To Examine the Horse's Larynx . . . . . . . . 137

SOCIETY PROCEEDINGS . . . . . . . . 138
VETERINARY COLLEGE NOTES . . . . . . . . 139
NECROLOGY . . . . . . . . 140
REVIEW DEPARTMENT . . . . . . . . 141
BOOKS AND PAMPHLETS RECEIVED . . . . . . . . 142

